# How Family Functioning Shapes Adolescent Adjustment: The Mediating Role of Interpersonal Competence

**DOI:** 10.3390/bs15111441

**Published:** 2025-10-23

**Authors:** Yuhan Jiang, Leping Huang, Yi Song, Jingxin Wang, Kuo Zhang

**Affiliations:** 1 Faculty of Psychology, Tianjin Normal University, Tianjin 300382, China; noloft@126.com (Y.J.); huanglepingtuc@126.com (L.H.); emilyfate@163.com (Y.S.); wjxpsy@126.com (J.W.); 2 School of Foreign Language, Tianjin University of Commerce, Tianjin 300134, China; 3 Department of Social Psychology, Nankai University, Tianjin 300071, China

**Keywords:** family functioning, interpersonal competence, adolescent adjustment, social and emotional learning (SEL), mediating effect

## Abstract

Adolescence is a critical stage of emotional and social development, with family functioning playing a vital role in shaping adolescent adjustment. However, the mechanisms linking family functioning to adolescent adjustment, particularly the mediating role of interpersonal competence in China, remain underexplored. This study surveyed 7318 junior and senior high school students from multiple Chinese regions, assessing family cohesion, family adaptability, interpersonal competence (communication, regulation, perception), and adolescent adjustment. Regression and mediation analyses examined direct and indirect effects of family functioning on adjustment. Among junior high students, family cohesion indirectly influenced adjustment via communication (28.10%) and regulation (17.32%), while adaptability operated through communication (29.50%) and regulation (32.45%). Among senior high students, cohesion acted via communication (18.63%) and regulation (21.57%), whereas adaptability affected adjustment equally through both (31.29%). Findings reveal developmental stage differences in the relative importance of interpersonal competence dimensions, confirm the applicability of the Social and Emotional Learning (SEL) framework in China, and provide evidence for stage-specific interventions to strengthen interpersonal skills and optimize family–school support systems for adolescent adjustment.

## 1. Introduction

In the post-pandemic era, mental health issues of middle school students have become increasingly prominent, attracting widespread attention from society, schools, and families, as well as ongoing interest from fields such as psychology, sociology, and education. Previous studies suggest that mental health problems among students will not disappear overnight after the pandemic but will accumulate over time (Shek, 2024). Unfortunately, despite the critical importance of maintaining mental health during and after the pandemic, many regions around the world have failed to adequately address mental health concerns. According to a survey by the World Health Organization (WHO), approximately 30% of adolescents reported that the COVID-19 pandemic had a negative impact on their mental health, primarily manifesting in difficulties with school adjustment, academic decline, and social isolation (World Health Organization, 2023). Adolescence (12–18 years) marks the transition from childhood to adulthood (Erikson, 1966). This period is characterized by profound changes across biological, psychological, and social domains, alongside heightened exposure to academic stress, interpersonal challenges, and emotional lability (Fan & Sun, 2013). There is an increasing trend of mental health issues becoming more prevalent and emerging at younger ages (Huang et al., 2022). Studies show that adolescents with lower mental health literacy exhibit significant differences from their peers in emotional regulation, stress coping, and interpersonal communication (D. Liu, 2024), which are closely related to their adjustment abilities (Cobos-Sánchez et al., 2017). Without timely intervention, these differences may lead to more serious psychological and behavioral problems (Yu & Li, 2021).

Given the increasing prevalence of mental health problems among adolescents, particularly in the post-pandemic context, research should focus not only on symptomatology but also on adolescents’ broader adaptive capacity (Maynard et al., 2025). Adolescent adjustment is defined as the ability of individuals to form psychological and behavioral patterns necessary for fulfilling social life (Xu, 2000). More specifically, it refers to adolescents’ capacity to respond to changes occurring during the process of socialization and to achieve congruence with their environment (Zhang & Jiang, 2006). Adolescent adjustment encompasses psychological adjustment (e.g., individual’s psychological and behavioral responses to their environment, reflecting the capacity to actively regulate emotions and exercise self-control; Sekar & Lawrence, 2016), behavioral adjustment (e.g., ongoing self-regulation to adapt to environmental and learning demands, maintaining alignment with the surrounding context; Feng & Li, 2002), and social adjustment (e.g., the harmony between the individual and their social environment, including emotional well-being and the internalization of social values; Paris & White-Williams, 2005). In this study, we further define adjustment as the specific psychological and behavioral responses that arise when individuals are influenced by external environments and grounded in their physical and psychological experiences. In other words, adjustment can be considered the external manifestation of psychological change, and in the turbulent context of the post-pandemic era, it is closely related to mental health (Campione-Barr et al., 2021).

In understanding the mechanisms that influence adolescent adjustment, family functioning, as a core element of early socialization, plays a profound role in shaping psychological development and adjustment abilities (Liao et al., 2023; Shek, 2002). Additionally, interpersonal competence, which includes emotional understanding, communication, and cooperation skills in adolescents’ interactions, may mediate the relationship between family functioning and adolescent adjustment. Strong interpersonal competence, as a key component of socio-emotional competence, not only helps establish positive relationships and enhance social acceptance but also boosts adolescents’ confidence and ability to face environmental challenges (Lin et al., 2024; Martinez & Gomez, 2024). The United Nations Educational, Scientific and Cultural Organization (UNESCO) emphasizes in the Education 2030 Framework that the development of children’s emotional cognition and social communication skills should be integrated into core educational goals (UNESCO, 2016). Against this backdrop, investigating the relationship between family functioning, interpersonal competence, and adolescent adjustment is not only of significant theoretical importance but also provides practical implications for optimizing educational intervention strategies and promoting the mental health development of middle school students.

### 1.1. Family Functioning and Adolescent Adjustment

Bronfenbrenner’s (1979) Ecological Systems Theory emphasizes the profound influence of the family, as a core microsystem, on an individual’s psychological and social adjustment. The role of family functioning in adolescent adjustment has also been supported by Shek’s research (Shek, 2002). Family functioning is a multidimensional, comprehensive concept that includes the relational structure among family members, communication quality, emotional bonds, and the ability to cope with environmental changes (Beavers & Hampson, 2000; D. H. Olson, 2000). Among these, family cohesion reflects the strength of emotional bonds between family members and serves as the core of mutual recognition, support, and emotional exchange (Beavers & Hampson, 2000; D. H. Olson, 2000; J. Tang & Jiang, 2009). Family adaptability refers to the ability of family members to flexibly adjust family rules and internal norms in response to external environmental changes and developmental challenges (Fei et al., 1991). Adjustment is the ability of individuals to form corresponding psychological and behavioral patterns to fulfill the demands of social life (Xu, 2000). Zhang and Jiang (2006) defined adolescent adjustment as an individual’s capacity to adjust to changes occurring during the process of socialization, thereby achieving congruence with the surrounding environment. Positive family functioning not only provides emotional support and social resources to adolescents but also fosters the development of their socio-emotional competencies, enhancing their interpersonal communication, emotional management, and social responsibility, which significantly improves their adolescent adjustment ability (Shek, 2002). Conversely, inadequate family functioning often leads to decreased self-esteem, increased mental health issues, and difficulties in forming stable peer relationships, resulting in poor adjustment (Yen et al., 2013). Research shows that stronger family cohesion and adaptability provides more positive support for adolescents, facilitating emotional and social development and promoting positive behavioral adjustment (Choi & Becher, 2019; Shao et al., 2018). In contrast, a negative or weak emotional connection in the family environment can increase adolescent feelings of loneliness, reduce social responsibility, and lead to adjustment difficulties (Du, 2023). Therefore, this study proposes Hypothesis 1: Family functioning positively predicts adolescent adjustment level.

### 1.2. Family Functioning and Interpersonal Competence

Interpersonal competence, also referred to as interpersonal communication competence, is a key psychological quality that enables individuals to build and maintain high-quality interpersonal relationships through effective interaction with others or groups (Hao & Niu, 2015; X. Liu et al., 2016). It typically comprises three dimensions: interpersonal communication, interpersonal perception, and interpersonal regulation (Shen et al., 2009). Interpersonal communication refers to the exchange of information and psychological signals, serving as a motivational force that meets individual needs, and is regarded as a form of social intelligence (Rogers & Koch, 1959; Wentzel, 1998). Interpersonal perception involves recognizing and understanding others’ appearances, personalities, and behaviors, falling within the domain of social cognition (Guo et al., 2017). Negative interpersonal perception may lead to emotional withdrawal and maladaptive social interactions, thereby undermining the quality of real-life relationships (Sadikaj et al., 2017). Interpersonal regulation refers to the ability to flexibly apply communication strategies to manage and adjust interpersonal relationships, which is essential for effective interaction (Ma & Bai, 2006; Shi, 2013).

As a critical distal environmental factor influencing adolescent psychological and social development, family functioning plays an essential role in shaping interpersonal competence. Healthy family functioning, characterized by high levels of cohesion and adaptability, provides adolescents with stable emotional support and a sense of security, thereby fostering the development of interpersonal communication skills (Dai, 2021; Shek, 2002). Studies have shown that harmonious family relationships not only contribute to adolescents’ resilience and optimism but also help reduce behavioral problems (Sonego et al., 2013; Yang, 2019). Furthermore, parental education and attention to parent–child relationships enhance the positive impact of family functioning on adolescents’ emotional intelligence and interpersonal communication skills (Li et al., 2024). In contrast, dysfunctional family environments often lead to emotional detachment among members, decreased interpersonal trust, and lower communication quality, making adolescents more likely to adopt maladaptive interpersonal coping strategies, thereby negatively affecting their social adjustment (Geng et al., 2021). Additionally, positive family functioning promotes secure parent–child attachment and facilitates the development of positive cognitive schemas, providing a strong foundation for healthy peer relationships and significantly influencing interpersonal behavior (D. Zhao et al., 2019).

In summary, through emotional warmth and effective communication, family functioning plays a significant role in promoting the development of adolescents’ interpersonal competence. Based on this, the present study proposes Hypothesis 2a: Family functioning positively predicts the level of interpersonal competence in adolescents.

### 1.3. Interpersonal Competence and Adolescent Adjustment

In adolescent development research, interpersonal competence has been shown to be closely related to well-being, emotional regulation, and school adjustment (Geng et al., 2021; Lee & Yun, 2020). Good adjustment abilities help individuals establish positive interpersonal relationships, improve relationship quality, and enhance their willingness and ability to engage in social activities (C. Liu et al., 2025). As adolescents improve the quality of their interpersonal relationships, their interpersonal needs are more easily met, which in turn promotes better adjustment to the constantly changing external environment (Vorauer et al., 2020). Existing studies have shown that interpersonal communication skills can significantly and positively predict new students’ adjustment, while negative communication motives can lower individuals’ adjustment levels in both psychological and school-related domains (Y. Chen et al., 2019). Based on these findings, this study proposes hypothesis H2b: Interpersonal competence positively predicts adolescent adjustment.

### 1.4. The Mediating Role of Interpersonal Competence

Previous research has indicated that family functioning plays a significant role in adolescent adjustment (T. Tang et al., 2025). However, this effect is not entirely direct, and it may involve key psychological mechanisms. According to Bronfenbrenner’s (1979) ecological systems theory, individual development is influenced by multiple nested systems, with the family, as the most central microsystem, indirectly affecting adjustment outcomes through its impact on social behavior and psychological capabilities. Meanwhile, interpersonal competence, as a key factor in social adjustment, is regarded as the foundation for an individual’s successful adjustment to the social environment (Vorauer et al., 2020).

The Collaborative for Academic, Social, and Emotional Learning (CASEL) model has been recognized and widely implemented in various educational environments globally. It identifies five key competencies: self-awareness, social awareness, self-management, relationship skills, and responsible decision-making (Majolo et al., 2023). The development of these competencies is rooted in early interpersonal interactions and supportive environments within the family (Srivastva & Ramanujan, 2019). By fostering non-cognitive skills such as interpersonal communication, providing quality early childhood education has become a fundamental goal for many developed countries in achieving 21st-century skills (Hayashi et al., 2022).

It is noteworthy that the pathways through which family functioning influences adolescent development may vary across cultural contexts. In Western societies, where individual independence and school system autonomy are more prominent, peer relationships and school structures are often seen as important external factors for adolescent adjustment (Eccles & Roeser, 2005). In Eastern cultures, especially in traditional Chinese society, the family not only provides emotional support and behavioral regulation but is also viewed as the core starting point for socialization and a continuous source of influence on individuals (C.-C. D. Wang & Mallinckrodt, 2006). Therefore, exploring the mechanisms of the SEL framework in Eastern cultures is especially important. Based on this, the present study proposes that interpersonal competence mediates the relationship between family functioning and adolescent adjustment.

### 1.5. The Current Study

Social and Emotional Learning (SEL), as a professional term in the field of education, along with its related curriculum design and implementation, remains a relatively new area of exploration in China (X. Wang, 2022). Social and emotional factors play a crucial role in students’ growth, but how to improve and implement the SEL concept still requires in-depth consideration. Indeed, the recent expansion of SEL practices in the United States, particularly in school curricula and family education interventions (Sandilos et al., 2023; Skaar & Townsley, 2025), provides valuable experience for us. However, when drawing on these experiences, it is essential first to understand the cultural background in which the concept is rooted and how it shapes its core ideas, followed by a profound and reflective examination. Compared to Western families, which emphasize the promotion of individual independence, Chinese families place more emphasis on attachment and relationality, making it more worth exploring the potential impact of family functioning on adolescents’ interpersonal competence and adolescent adjustment. In the present study, we focus specifically on relationship skills, a core SEL competency, as it directly captures adolescents’ ability to establish and maintain positive interpersonal relationships, which is highly relevant for adolescent adjustment.

Moreover, we analyzed junior and senior high school students separately, due to the developmental differences between these two age groups. Junior high school students are generally in early adolescence, a stage marked by rapid changes in physiology, cognition (Steinberg, 2005), during which their cognitive abilities, interpersonal competence, and self-regulation are still developing (Brown & Larson, 2009). In contrast, most senior high school students tend to be in middle-to-late adolescence, when cognitive maturity and self-regulation increase (Steinberg & Morris, 2001), but students also encounter more complex interpersonal relationships, heavier academic stress, and greater challenges in identity formation and future planning (Yuan et al., 2025). Thus, separate analyses allow for a more accurate examination of these potentially distinct developmental patterns.

Therefore, the core pathway of this study is to investigate whether family functioning influences adolescent adjustment by promoting interpersonal competence in their social-emotional abilities. Based on the theoretical foundation and cultural context, the following hypotheses are proposed:

**H1:**  *Family cohesion and family adaptability positively predict adolescent adjustment.*

**H2a:**  *Family cohesion and adaptability positively predict adolescents’ interpersonal competence and its dimensions.*

**H2b:**  *Each dimension of interpersonal competence positively predicts adolescent adjustment.*

**H3:**  *The dimensions of interpersonal competence mediate the relationship between family functioning and adolescent adjustment.*

## 2. Materials and Methods

### 2.1. Participants and Procedure

After obtaining ethical approval from the institution of the first author, the research team conducted an online survey with middle school students from over ten schools in Tianjin, Shandong, Inner Mongolia, Shanghai, Guizhou, Jiangxi, and other provinces in China. Before completing the survey, all participants were informed about the purpose and procedures of the study. They were given the right to provide informed consent and withdraw at any time. The surveys were completed at school and collected immediately after completion. Written informed consent was signed by all participants’ guardians. A convenience sampling method was used, with a total of 7788 questionnaires distributed. After excluding invalid responses, 7318 valid questionnaires were obtained, resulting in a valid response rate of 93.97%. Among the participants, 3351 were male (45.8%) and 3967 were female (54.2%). In this study, 4258 were junior high school students (M age = 14.40, SD = 1.00) and 3060 were senior high school students (M age = 16.73, SD = 0.75).

### 2.2. Measures

#### 2.2.1. Family Adaptability and Cohesion Evaluation Scales II (FACES II-CV)

The Chinese version of the Family Adaptability and Cohesion Evaluation Scales II (FACES II), revised by Fei et al. (1991) and originally developed by D. Olson (1989), was used in this study. The scale consists of 30 items, with two dimensions: cohesion and adaptability. Responses were rated on a 5-point Likert scale (from “Never” to “Always”). Sample items for the cohesion dimension include: “When conflicts arise in the family, family members keep their thoughts to themselves.” Sample items for the adaptability dimension include: “At home, we do things together.” In this study, the Cronbach’s alpha for the Family Cohesion and Adaptability Scale was 0.93, with alpha values of 0.87 for cohesion and 0.85 for adaptability.

#### 2.2.2. Adjustment Scale for Adolescents (ASA)

The Adjustment Scale for Adolescents (ASA) is used to assess students’ overall adolescent adjustment (Zhang & Jiang, 2006). The scale contains 22 items, with responses ranging from “Completely Disagree” to “Completely Agree,” and higher scores indicating a higher level of adolescent adjustment. Sample items include: “In daily life, I can display emotions appropriate to the situation” and “I do not feel nervous about the changes brought by physical development.” The scale showed excellent internal consistency in this study, with a Cronbach’s alpha of 0.95, indicating high reliability.

#### 2.2.3. Interpersonal Competence Scale

The Interpersonal Competence Scale is employed to assess adolescents’ ability to communicate and interact effectively in their daily lives (Ma & Bai, 2006). The scale includes three dimensions: interpersonal communication ability, interpersonal perception, and interpersonal regulation, with a total of 16 items. Responses ranged from “Completely Disagree” to “Completely Agree,” with higher scores indicating better interpersonal competence. Sample items include: “I can make many friends because I get along well with others” (interpersonal communication), “When conflicts arise, I can properly adjust my strategies to maintain good relationships” (interpersonal regulation), and “I can quickly and accurately understand others’ intentions when I get to know them” (interpersonal perception). The Cronbach’s alpha for the Interpersonal Competence Scale was 0.935, with alpha values of 0.912 for communication ability, 0.896 for perception, and 0.802 for regulation.

### 2.3. Data Analysis

Data were analyzed using SPSS 27.0 software for descriptive statistics, correlation analysis, hypothesis testing, and regression analysis. The statistical processing of the questionnaire data revealed the relationships between variables. Mediation effects were tested using the PROCESS V6.5 plugin, with significance tests for indirect effects. Additionally, Amos 24.0 software was used to test the mediation effects and construct the SEM model to verify the path relationships and model fit. Based on the above analysis, the study quantitatively explored the internal relationships between family functioning, interpersonal competence, and adolescent adjustment in middle school students.

## 3. Results

### 3.1. Common Method Bias Test

Harman’s single-factor test was conducted to examine potential common method variance. An unrotated exploratory factor analysis identified eight factors with eigenvalues greater than 1. The first factor accounted for 37.54% of the total variance, which is below the critical threshold of 40%, suggesting that common method bias was effectively minimized in the present study and is unlikely to have substantially influenced the results.

### 3.2. Correlation Analysis

Pearson product–moment correlation analysis was conducted to examine the relationships between family functioning (cohesion and adaptability) and interpersonal competence as well as its dimensions. The results are presented in Table 1.

Descriptive statistics and correlations of the variables are shown in Table 1. The results indicated that the dimensions of family functioning, interpersonal competence, and adolescent adjustment were all significantly positively correlated.

### 3.3. Mediating Role of Interpersonal Competence in the Relationship Between Family Functioning and Adolescent Adjustment Among Junior High School Students

Following the stepwise regression procedure recommended by Wen and Ye (2014), the mediating role of interpersonal competence was examined. The results indicated that multiple dimensions of interpersonal competence mediated the relationship between family functioning (cohesion and adaptability) and adolescent adjustment among junior high school students (see Table 2).

Step 1 of the hierarchical regression model showed that, after controlling relevant demographic variables, both family cohesion (*β* = 0.31, *p* < 0.001) and family adaptability (*β* = 0.34, *p* < 0.001) significantly and positively predicted adolescent adjustment among junior high school students.

Step 2 results indicated that, except for the non-significant predictive effect of family cohesion on interpersonal perception, both family cohesion and family adaptability significantly and positively predicted all dimensions of interpersonal competence, explaining approximately 26% of the variance in interpersonal communication, 26% in interpersonal regulation, and 10% in interpersonal perception. The predictive effects of family adaptability on these dimensions (*β* = 0.27–0.36, *ps* < 0.001) were stronger than those of family cohesion (*β* = 0.17–0.24, *ps* < 0.001).

Step 3 results revealed that all dimensions of interpersonal competence (interpersonal communication, interpersonal perception, and interpersonal regulation) significantly and positively predicted adolescent adjustment. Moreover, the predictive effects of family cohesion and adaptability on adolescent adjustment remained significant, suggesting that these dimensions of interpersonal competence mediated the relationship between family cohesion/adaptability and adolescent adjustment among junior high school students.

Based on these results, the following path model was constructed to illustrate the relationships among family functioning, interpersonal competence, and adolescent adaptation in junior high school students.

**Figure 1:**
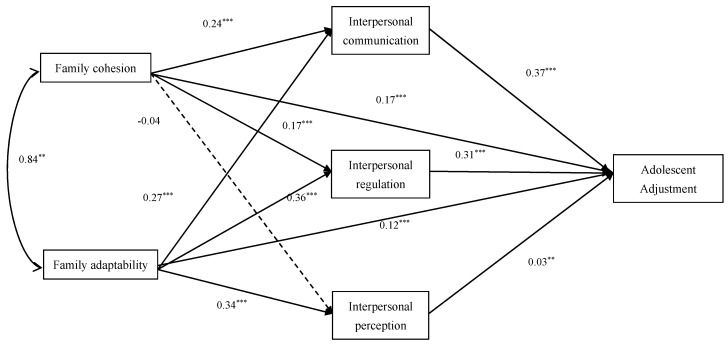
Path model of the relationships among family functioning, interpersonal competence, and adolescent adjustment (junior high school students). Note. ** *p* < 0.01, *** *p* < 0.001.

From the path model in Figure 1, it can be concluded that both family cohesion and family adaptability significantly predict adolescent adjustment among junior high school students. In addition, family cohesion and family adaptability influence all dimensions of interpersonal competence (except for the non-significant effect of family cohesion on interpersonal perception) and, through interpersonal competence, indirectly affect adolescent adjustment. The model further indicates that, even after incorporating multiple dimensions of interpersonal competence as mediating variables, the predictive effects of family cohesion and family adaptability on adolescent adjustment remain significant. This suggests that the various dimensions of interpersonal competence play a partial mediating role in the relationship between family functioning and adolescent adjustment, thereby shaping the association between the two.

Using the PROCESS macro, we examined the mediating effects of the three dimensions of interpersonal competence—interpersonal communication, interpersonal regulation, and interpersonal perception—on the relationships between family cohesion/adaptability and adolescent adjustment among junior high school students. Standardized indirect effect sizes, 95% confidence intervals (CIs), and the proportion of the total effect explained by each indirect path are reported in Table 3.

For family cohesion, the total indirect effect via all three interpersonal competence dimensions was statistically significant, *β* = 0.138, 95% CI [0.108, 0.169], accounting for 45.10% of the total effect. Specifically, the indirect effect through interpersonal communication (*β* = 0.086, 95% CI [0.068, 0.107]) accounted for 28.10% of the total effect, and through interpersonal regulation (*β* = 0.053, 95% CI [0.037, 0.069]) accounted for 17.32%. The indirect effect through interpersonal perception was negligible and non-significant (*β* = −0.001, 95% CI [−0.004, 0.000]), contributing only 0.33% of the total effect.

For family adaptability, the total indirect effect via interpersonal competence was also significant, *β* = 0.222, 95% CI [0.190, 0.255], explaining 65.49% of the total effect. The indirect effect through interpersonal communication (*β* = 0.100, 95% CI [0.080, 0.120]) accounted for 29.50%, and through interpersonal regulation (*β* = 0.110, 95% CI [0.092, 0.130]) accounted for 32.45% of the total effect. The indirect effect via interpersonal perception was small but significant (*β* = 0.012, 95% CI [0.004, 0.020]), accounting for 3.54% of the total effect.

These findings suggest that, for junior high school students, interpersonal communication and interpersonal regulation are the primary mediating pathways linking both family cohesion and adaptability to adolescent adjustment, while interpersonal perception plays a minimal role.

### 3.4. Mediating Role of Interpersonal Competence in the Relationship Between Family Functioning and Adolescent Adjustment Among Senior High School Students

Following the stepwise regression procedure recommended by Wen and Ye (2014), the mediating role of interpersonal competence was examined. The results indicated that multiple dimensions of interpersonal competence mediated the relationship between family functioning (family cohesion and family adaptability) and adolescent adjustment among senior high school students (see Table 4).

Step 1 of the hierarchical regression model showed that, after controlling relevant demographic variables, both family cohesion (*β* = 0.20, *p* < 0.001) and family adaptability (*β* = 0.44, *p* < 0.001) significantly and positively predicted adolescent adjustment among senior high school students.

Step 2 results indicated that family cohesion and family adaptability significantly and positively predicted all dimensions of interpersonal competence, explaining approximately 24% of the variance in interpersonal communication, 25% in interpersonal regulation, and 12% in interpersonal perception. The predictive effects of family adaptability on these dimensions (*β* = 0.39–0.42, *ps* < 0.001) were stronger than those of family cohesion (*β* = 0.11–0.12, *ps* < 0.001).

Step 3 results revealed that all dimensions of interpersonal competence (interpersonal communication, interpersonal perception, and interpersonal regulation) significantly and positively predicted adolescent adjustment. Moreover, the predictive effects of family cohesion and family adaptability on adolescent adjustment remained significant, suggesting that these dimensions of interpersonal competence mediated the relationship between family cohesion/adaptability and adolescent adjustment among senior high school students.

Based on these results, the following path model was constructed to illustrate the relationships among family functioning, interpersonal competence, and adolescent adjustment in senior high school students.

**Figure 2:**
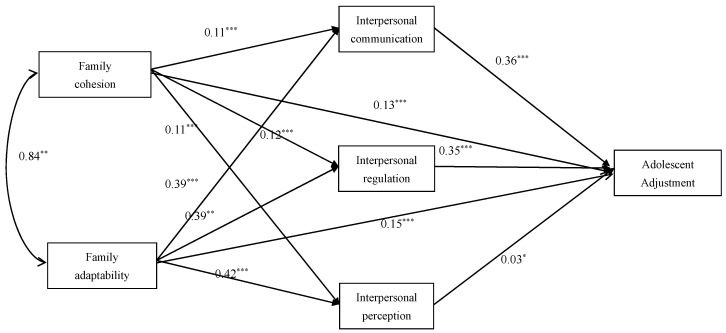
Path model of the relationships among family functioning, interpersonal competence, and adolescent adjustment (senior high school students). Note. * *p* < 0.05. ** *p* < 0.01. *** *p* < 0.001.

As shown in Figure 2, both family cohesion and family adaptability significantly predicted adolescent adjustment among senior high school students. They also influenced all dimensions of interpersonal competence (interpersonal communication, interpersonal perception, and interpersonal regulation), which in turn indirectly affected adolescent adjustment. The model further indicated that, even after including these dimensions of interpersonal competence as mediators, family cohesion and family adaptability remained significant positive predictors of adolescent adjustment, suggesting partial mediation by the dimensions of interpersonal competence in the relationship between family functioning and adolescent adjustment.

For family cohesion, the total indirect effect via all three interpersonal competence dimensions was statistically significant, *β* = 0.078, 95% CI [0.039, 0.117], accounting for 38.24% of the total effect. Specifically, the indirect effect through interpersonal communication (*β* = 0.038, 95% CI [0.016, 0.060]) accounted for 18.63% of the total effect, and through interpersonal regulation (*β* = 0.044, 95% CI [0.022, 0.065]) accounted for 21.57%. The indirect effect through interpersonal perception was negligible and non-significant (*β* = −0.003, 95% CI [−0.008, 0.000]), contributing only 1.47% of the total effect (see Table 5).

For family adaptability, the total indirect effect via interpersonal competence was also significant, *β* = 0.289, 95% CI [0.248, 0.330], explaining 65.53% of the total effect. The indirect effects through interpersonal communication (*β* = 0.138, 95% CI [0.113, 0.164]) and interpersonal regulation (*β* = 0.138, 95% CI [0.115, 0.164]) each accounted for 31.29% of the total effect, while the indirect effect via interpersonal perception (*β* = 0.013, 95% CI [0.001, 0.026]) accounted for 2.95% of the total effect (see Table 5).

These findings suggest that, for senior high school students, interpersonal communication and interpersonal regulation are the primary mediating pathways linking both family cohesion and adaptability to adolescent adjustment.

## 4. Discussion

This study used a large-scale questionnaire survey with middle school students, employing the FACES II-CV, the Interpersonal Competence Scale, and the Adjustment Scale for Adolescents to examine the relationships between Family Cohesion, Family Adaptability, Interpersonal Competence, and Adolescent Adjustment. Grounded in the SEL framework, the findings highlight the mediating role of Interpersonal Competence in linking Family Functioning to adolescents’ adjustment outcomes.

### 4.1. Relationship Between Family Functioning and Adolescent Adjustment

For both junior and senior high school students, family cohesion showed a significant positive predictive effect on social adjustment, indicating that the closeness among family members can influence the degree of an individual’s socialization (Hawkins, 1996). In Eastern cultural contexts, the family is typically regarded as the core unit of socialization, providing adolescents with emotional support and a stable social environment. High family cohesion fosters strong emotional bonds among members, enabling individuals to actively seek guidance and assistance from family when facing external crises or emotional fluctuations, thereby effectively coping with challenges and enhancing adjustment (Gross, 2015).

In addition, family adaptability was also found to be significantly associated with adolescent adjustment. Reductions in family cohesion and adaptability may lead to a lack of interaction with parents and insufficient emotional support for adolescents (Mierjiang, 2024; Yang, 2018). In the Chinese cultural context, emotional connections among family members are considered fundamental to adolescents’ mental health and social adjustment. The absence of such support may hinder adolescents’ abilities in managing interpersonal relationships, understanding social roles, self-evaluation, and forming expectations and trust toward others, ultimately undermining their adjustment capacity (Chi et al., 2021).

Overall, family functioning plays a clear predictive role in students’ adaptive behaviors. A well-functioning family system can effectively prevent adjustment problems and foster healthy development in adolescents.

### 4.2. The Mediating Role of Interpersonal Competence in Family Functioning and Adolescent Adjustment Among Junior High School Students

The present study revealed that interpersonal competence serves as a significant mediator in the relationship between family functioning and adolescent adjustment among junior high school students. Specifically, the total indirect effect of family intimacy was 0.138, accounting for 45.10% of the total effect. This effect primarily operated through interpersonal communication skills (28.10%) and interpersonal regulation skills (17.32%), whereas the mediating role of interpersonal perception was negligible (0.33%). These results are consistent with the findings of Y. Liu et al. (2023), suggesting that during early adolescence, emotional support and close familial relationships play a crucial role in facilitating students’ adjustment to the school environment. A high-cohesion family environment can provide junior high school students with stable emotional support and a sense of security, enabling them to communicate more proactively, coordinate relationships, and effectively manage their behavior when facing peer conflicts and academic stress (Zhou et al., 2023). Interpersonal communication skills contributed the most to the mediating effect, as developing strong social skills is essential for students’ successful integration into the school environment (X. Chen et al., 2000). At the same time, the role of interpersonal regulation skills should not be underestimated, as they help students adjust their behavior and maintain positive interactions in situations involving emotional fluctuations or conflicts, thereby further enhancing their adjustment.

For junior high school students, the total indirect effect of family adaptability was even higher (0.222, accounting for 65.49% of the total effect), primarily reflected in the enhancement of interpersonal communication skills (29.50%) and interpersonal regulation skills (32.45%). When facing academic pressure, peer competition, and changes in the social environment, the flexibility and problem-solving capacity of the family can provide critical support, making it easier for adolescents to adapt to school and social life (J. Tang & Jiang, 2009; X. Zhao, 2020). When families can adjust rules and expectations according to adolescents’ needs and situational demands, and provide strategies for resolving conflicts and overcoming difficulties, they can facilitate the application of interpersonal communication skills in real-life social contexts (Ke & Yang, 2025).

Overall, the role of interpersonal communication in junior high school students was slightly stronger than that of interpersonal regulation, suggesting that the establishment of social skills is particularly important for their successful integration into the school environment. Early adolescence is a critical period for the development of interpersonal skills, and immature communication strategies coupled with weaker interpersonal regulation abilities can easily lead to difficulties in social adjustment (Y. Chen et al., 2019). This stage also represents an important period for social and emotional development Aspen Institute National Commission on Social, Emotional, and Academic Development, 2018), yet research and interventions in social and emotional learning (SEL) targeting this unique developmental stage remain relatively scarce, particularly compared with those in primary school. At the societal level, early adolescence is a time when peer relationships become increasingly significant (Destin et al., 2018). Adolescents seek a sense of belonging within both peer and adult groups and begin to explore identity and social status in these contexts (Deutsch et al., 2017). Peer recognition and feedback can have a substantial influence on their sense of self-worth and behavioral choices (Devaney et al., 2018). Therefore, in early adolescence, strong communication and collaboration skills play a more critical role in adjustment, which also explains why interpersonal communication exerts a more prominent mediating effect between family functioning and adolescent adjustment. The weaker effect of interpersonal perception may be attributed to the fact that junior high school students’ self-awareness and social awareness are not yet fully developed, limiting their capacity for social perception.

### 4.3. The Mediating Role of Interpersonal Competence in Family Functioning and Adolescent Adjustment Among Senior High School Students

For senior high school students, the total indirect effect of family cohesion was lower than that observed for junior high school students (0.078, accounting for 38.24% of the total effect). Interpersonal communication (18.63%) and interpersonal regulation (21.57%) remained the primary pathways, while the mediating role of interpersonal perception was still negligible (1.47%). This pattern suggests that as adolescents mature, they become more independent and less emotionally reliant on their families, thereby diminishing the impact of family cohesion on their adjustment.

In contrast, the total indirect effect of family adaptability in senior high school students remained relatively high (0.289, accounting for 65.53% of the total effect), exerting its influence on adolescent adjustment mainly through interpersonal communication and interpersonal regulation (both at 31.29%). Notably, interpersonal regulation emerged as a more critical mediator between family functioning and adolescent adjustment in this age group. Faced with mounting academic demands, evolving social roles, and increasingly complex interpersonal dynamics, senior high school students tend to rely more on their families’ flexibility and adaptability to support emotional regulation (Xia et al., 2022). Highly adaptable families can help adolescents manage their emotions and behavior, reduce social anxiety (Xia et al., 2022), and strengthen their social communication skills—thereby facilitating adolescent adjustment and promoting overall psychological well-being. During this developmental stage, adolescents are required to demonstrate stronger emotional regulation and conflict resolution skills (Yuan et al., 2025), rather than depending solely on relational skills. This may explain why interpersonal regulation plays a more prominent mediating role in linking family function to positive adolescent adjustment among senior high school students.

An additional noteworthy finding was that family adaptability was significantly negatively associated with interpersonal perception among senior high school students—a relationship opposite to that observed in junior high school students. This may be related to the higher prevalence of internet and mobile phone dependence in this older group. Prior research has shown that individuals with internet dependence often exhibit lower nonverbal sensitivity and weaker interpersonal perception (Guo et al., 2017). Consistent with this, Q. Wang et al. (2018) found that high school students scored significantly higher on mobile phone dependence than junior high school students. Moreover, large-scale survey data have shown that the prevalence of Internet addiction increases with age, indicating that internet and mobile phone dependence tends to intensify across higher grade levels (Yan et al., 2022). Given that senior high school students generally have more frequent exposure to electronic devices, those in well-functioning families with greater autonomy may be more susceptible to such dependency, which in turn could impair the accuracy of their social perception.

Building upon these findings, this study draws on the ecological systems and SEL frameworks to interpret how family functioning influences adolescent adjustment through interpersonal competence. Unlike previous research that has primarily focused on the link between family functioning and adolescent adjustment, the present study makes an innovative contribution by integrating the construct of interpersonal competence, examining its mediating role in the relationship between family functioning and adolescent adjustment. This approach reveals the underlying mechanisms through which family functioning influences adolescent adjustment. Drawing on ecological systems theory and the social and emotional learning (SEL) framework, the study proposes a “family functioning–interpersonal competence–adolescent adjustment” model and, for the first time, systematically examines the distinct pathways through which family cohesion and family adaptability operate across different dimensions of interpersonal competence. This model moves beyond the single-perspective approaches common in prior research, offering a more comprehensive analytical framework that deepens our understanding of how the family environment can enhance emotional regulation and interpersonal communication skills, thereby improving adolescent adjustment. Identifying the specific dimensions of interpersonal competence that promote social and emotional learning in secondary school students can enable more targeted support, maximize the potential of SEL, and lay the foundation for healthy development and positive relationships—ultimately enhancing academic, career, and life success.

### 4.4. Practical Implications

An integrated comparison of the pathways for junior and senior high school students reveals that interpersonal competence, as a mediating variable, demonstrates different emphases across its dimensions in linking family functioning to adolescent adjustment. Interpersonal communication exerted a stronger influence on adolescent adjustment among junior high school students, whereas interpersonal regulation contributed more substantially to adolescent adjustment among senior high school students. This pattern may reflect differences in the social environments and situational demands faced by adolescents at different developmental stages. Junior high school students are likely to engage more frequently in interactions within the family and among peers (Eisenstein et al., 2019) and thus rely more heavily on emotional support from the family. In contrast, senior high school students face greater social competition and more complex demands for emotional regulation (Yuan et al., 2025), requiring stronger family support in the form of rule flexibility and problem-solving capacity to navigate the increasingly complex school and social contexts. These findings highlight the need for age-specific educational interventions and family support strategies that target the key dimensions of interpersonal competence most relevant to each developmental stage, thereby more effectively fostering adolescents’ adjustment capacity.

These findings highlight the need for age-specific educational and family support strategies that strengthen the key dimensions of interpersonal competence at each developmental stage. Families can foster open dialogue through regular meetings or parent–child conversations to enhance emotional connection. For junior high school students, schools may focus on communication-based SEL courses and peer mentoring, while for senior high school students, programs on emotion regulation and stress management such as mindfulness and cognitive–behavioral training may better promote adolescent adjustment. Overall, with joint family–school support, developing age-appropriate interpersonal competencies can more effectively facilitate adolescents’ adjustment.

## 5. Limitations and Future Research Directions

This study employed convenience sampling, which, while ensuring a relatively broad sample size, may limit the representativeness of the sample and the generalizability of the findings. The methodology relied primarily on self-report questionnaires, lacking triangulation through interviews, experiments, or other diverse methods, thereby constraining deeper exploration of causal relationships. The selection of variables and dimensions was not exhaustive, as not all potential influencing factors were included, and the sample was limited to secondary school students, which further restricts the generalizability of the conclusions. Future research should adopt multi-method approaches, include broader and more diverse samples, and refine mechanism analyses to advance theoretical development. In addition, future research employing longitudinal or experimental designs could further examine reciprocal or moderating mechanisms.

## 6. Conclusions

The findings indicate that family functioning positively predicts adolescent adjustment, with interpersonal competence—interpersonal communication for junior high students and interpersonal regulation for senior high students—partially mediating this relationship. These findings enrich the theoretical framework on how the family environment influences adolescent adjustment through the development of socio-emotional skills, and they provide empirical evidence to inform both social and family-level strategies aimed at fostering students’ healthy development.

## Figures and Tables

**Table 1:** Correlations between Family Functioning and Dimensions of Interpersonal Competence.

Variable	M	SD	1	2	3	4	5	6	7	8
1. Family cohesion	71.6	11.22	1							
2. Family adaptability	50.36	9.80	0.84 ***	1						
3. Family functioning	60.98	10.07	0.96 ***	0.95 ***	1					
4. Interpersonal competence	3.77	0.73	0.44 ***	0.49 ***	0.49 ***	1				
5. Interpersonal communication	3.69	0.91	0.45 ***	0.48 ***	0.49 ***	0.88 ***	1			
6. Interpersonal regulation	3.98	0.75	0.46 ***	0.50 ***	0.50 ***	0.89 ***	0.73 ***	1		
7. Interpersonal perception	3.65	0.87	0.25 ***	0.32 ***	0.29 ***	0.84 ***	0.56 ***	0.62 ***	1	
8. Adolescent Adjustment	3.87	0.73	0.59 ***	0.61 ***	0.62 ***	0.78 ***	0.76 ***	0.75 ***	0.52 ***	1

*N* = 7318. *** *p* < 0.001. All tests were two-tailed.

**Table 2:** Hierarchical Regression Analyses Testing the Mediating Role of Interpersonal Competence Between Family Functioning and Adolescent Adjustment (Junior High School Students).

Predictor	Step 1: Adolescent Adjustment *β*		Step 2: Interpersonal Communication *β*	Step 2: Interpersonal Regulation *β*	Step 2: Interpersonal Perception *β*	Step 3: Adolescent Adjustment *β*
Control variables						
Gender	0.08 ***	0.07 ***	0.19 ***	−0.03	0.03 *	0.03 **
Age	−0.06 ***	−0.02	0.01	0.01	0.03 *	−0.03 **
Hukou status	0.05 **	0.04 **	0.04 *	0.05 **	0.06 ***	0.01
Only-child status	0.03	0.03 *	0.03 *	−0.01	0.00	0.02
Parental education	0.12 ***	0.01	−0.02	−0.03 *	0.02	0.02 *
Predictors						
Family cohesion		0.31 ***	0.24 ***	0.17 ***	−0.04	0.17 ***
Family adaptability		0.34 ***	0.27 ***	0.36 ***	0.34 ***	0.12 ***
Mediators						
Interpersonal communication						0.37 ***
Interpersonal regulation						0.31 ***
Interpersonal perception						0.03 **
*R* ^2^	0.04	0.4	0.26	0.26	0.1	0.71
*R* ^2^_adj_	0.04	0.37	0.26	0.26	0.1	0.71
*F*	34.07 ***	408.90 ***	211.51 ***	208.19 ***	70.55 ***	1015.59 ***

Note. * *p* < 0.05. ** *p* < 0.01. *** *p* < 0.001. All tests were two-tailed.

**Table 3:** Mediating Effects of Interpersonal Competence Between Family Functioning and Adolescent Adjustment (Junior High School Students).

Predictor	Path	Standardized Effect	95% CI Lower	95% CI Upper	Proportion (%)
Family Cohesion	Total indirect effect of interpersonal competence	0.138	0.108	0.169	45.10%
	Cohesion → Interpersonal Communication → Adjustment	0.086	0.068	0.107	28.10%
	Cohesion → Interpersonal Regulation → Adjustment	0.053	0.037	0.069	17.32%
	Cohesion → Interpersonal Perception → Adjustment	−0.001	−0.004	0.000	0.33%
Family Adaptability	Total indirect effect of interpersonal competence	0.222	0.190	0.255	65.49%
	Adaptability → Interpersonal Communication → Adjustment	0.100	0.080	0.120	29.50%
	Adaptability → Interpersonal Regulation → Adjustment	0.110	0.092	0.130	32.45%
	Adaptability → Interpersonal Perception → Adjustment	0.012	0.004	0.020	3.54%

Notes. CI = confidence interval.

**Table 4:** Hierarchical Regression Analyses Testing the Mediating Role of Interpersonal Competence Between Family Functioning and Adolescent Adjustment (Senior High School Students).

Predictor	Step 1: Adolescent Adjustment *β*		Step 2: Interpersonal Communication *β*	Step 2: Interpersonal Regulation *β*	Step 2: Interpersonal Perception *β*	Step 3: Adolescent Adjustment *β*
Control variables						
Gender	0.08 ***	0.06 ***	0.08 ***	−0.03	0.03	0.04 ***
Age	0.01	0.01	0.00	−0.03	−0.04 *	0.02 *
Hukou status	0.01	0.04	0.00	0.00	0.03	0.00
Only-child status	−0.03	−0.03 *	−0.02	−0.03	−0.04 *	−0.02
Parental education	0.08 ***	0.01	0.02	0.00	0.02	0.00
Predictors						
Family cohesion		0.20 ***	0.11 ***	0.12 ***	0.11 ***	0.13 ***
Family adaptability		0.44 ***	0.39 ***	0.39 **	0.42 ***	0.15 ***
Mediators						
Interpersonal communication						0.36 ***
Interpersonal regulation						0.35 ***
Interpersonal perception						0.03 *
*R* ^2^	0.01	0.39	0.24	0.25	0.12	0.72
*R* ^2^_adj_	0.01	0.38	0.24	0.25	0.12	0.72
*F*	8.31 ***	282.71 ***	139.88 ***	147.72 ***	57.58 ***	800.98 ***

Note. * *p* < 0.05. ** *p* < 0.01. *** *p* < 0.001. All tests were two-tailed.

**Table 5:** Mediating Effects of Interpersonal Competence Between Family Functioning and Adolescent Adjustment (Senior High School Students).

Predictor	Path	Standardized Effect	95% CI Lower	95% CI Upper	Proportion (%)
Family Cohesion	Total indirect effect of interpersonal competence	0.078	0.039	0.117	38.24%
	Cohesion → Interpersonal Communication → Adjustment	0.038	0.016	0.060	18.63%
	Cohesion → Interpersonal Regulation → Adjustment	0.044	0.022	0.065	21.57%
	Cohesion → Interpersonal Perception → Adjustment	−0.003	−0.008	0.000	1.47%
Family Adaptability	Total indirect effect of interpersonal competence	0.289	0.248	0.330	65.53%
	Adaptability → Interpersonal Communication → Adjustment	0.138	0.113	0.164	31.29%
	Adaptability → Interpersonal Regulation → Adaptation	0.138	0.115	0.164	31.29%
	Adaptability → Interpersonal Perception → Adaptation	0.013	0.001	0.026	2.95%

Notes. CI = confidence interval.

## Data Availability

The datasets generated and analyzed during the current study are not publicly available due to ethical restrictions on sharing sensitive information of minor participants but are available from the corresponding author upon reasonable request.
